# Supplementary material for: Muscle glycogen unavailability and fat oxidation rate during exercise: Insights from McArdle disease
Source: J Physiol. 2022 Nov 29;601(3):551–66. doi: 10.1113/JP283743 (PMC10099855; doi:10.1113/JP283743)
Supplement: Supplementary file 1 — Statistical Summary Document [file TJP-601-551-s002.docx]

**Manuscript Title**: Muscle glycogen unavailability and fat oxidation rate during exercise: Insights from McArdle disease

**Authors**: Carlos Rodriguez-Lopez, Alfredo Santalla, Pedro. L Valenzuela, Alberto Real-Martínez, Mónica Villarreal-Salazar, Irene Rodriguez-Gomez, Tomàs Pinós, Alejandro Lucia, Ignacio Ara.

**Animal model used, if applicable:** Mouse: McArdle (p.R50*/p.R50*) mice vs. Control (WT, p.R50R/p.R50R) mice.

**Underlying hypothesis**: Fat oxidation rate at maximal and submaximal exercise intensities is greater in patients with McArdle disease compared with sex- and age-matched healthy controls. In addition, maximal fat oxidation rate occurs at higher relative exercise intenisites in McArdle patiens than their healthy counterparts. Finally, lipid mobilization and catabolism is greater in McArdle mice in comparison to wild-type mice.

**Definitions of ‘n’:**

Questions 1 to 8: number of participants in the specified group

Questions 9 and 10: number of animals from which tissue dissections were obtained

**Statistical summary table**:

| **Experimental question number** | **Finding/ conclusion** | **Experimental location/variable** | **Mean value** | **Standard Deviation** | **n** | **Exact P value** | **Figure/table in which data are presented** | **Units** | **Data comparisons** | **Statistical test** | **Any other experimental factors** | **Comments** |
| --- | --- | --- | --- | --- | --- | --- | --- | --- | --- | --- | --- | --- |
| **1. Is the cardiorrespiratory fitness lower in McArdle patients? (i.e., VO2peak)** | Yes. | Controls | 42.500 | 11.400 | 12 | **<0.0001** | Table 1 | ml·kg-1·min-1 | McArdle vs. Control | Student's T test, unpaired | - | - |
|  |  | McArdle | 24.700 | 4.000 | 9 |  |  |  |  |  |  | - |
| **2. Is the peak work capacity lower in McArdle patients?** | Yes. | Controls | 3.230 | 0.970 | 12 | **<0.0001** | Table 1 | Watts·kg-1 | McArdle vs. Control | Student's T test, unpaired | - | - |
|  |  | McArdle | 1.280 | 0.360 | 9 |  |  |  |  |  |  | - |
| **3. Is maximal fat oxidation rate higher in Mcardle patients?** | Yes | Controls (absolute) | 0.330 | 0.104 | 12 | **0.001** | Figure 4A | g·min-1 | McArdle vs. Control | ANCOVA | VO2peak | - |
|  |  | McArdle (absolute) | 0.533 | 0.123 | 9 |  |  |  |  |  |  | - |
|  |  | Controls (Relative to fat free mass) | 6.415 | 2.247 | 12 | **<0.0001** | Figure 4A | mg·kg-1·min-1 | McArdle vs. Control | ANCOVA | VO2peak | - |
|  |  | McArdle (Relative to fat free mass) | 11.112 | 2.186 | 9 |  |  |  |  |  |  | - |
| **4.Does the maximal fat oxidation rate occur at a higher relative exercise intensity in McArdle patients?** | Yes | Controls | 41.3 | 9.1 | 12 | **<0.0001** | Figure 4B | %VO2peak | McArdle vs. Control | Student's T test, unpaired | - | - |
|  |  | McArdle | 94.4 | 7.2 | 9 |  |  |  |  |  | - | - |
| **5. Do McArdle patients have an increased work capacity using fat oxidation as the main energy substrate?** | Yes | Controls | 0.808 | 0.536 | 12 | **0.020** | Figure 4C | Watts·kg-1 | McArdle vs. Control | Student's T test, unpaired | - | - |
|  |  | McArdle | 1.330 | 0.348 | 9 |  |  |  |  |  | - | - |
| **6. Is the fat oxidation rate of McArdle patients higher during low to moderate relative exercise intensities?** | No. Fat oxidation rate are comparable to controls for the same relative exercise intensity | Controls (30% VO2peak) | 0.280 | 0.070 | 12 | **0.010** | Figure 3C | g·min-1 | McArdle vs. Control | Student's T test, unpaired | - | - |
|  |  | McArdle (30% VO2peak) | 0.152 | 0.076 | 9 |  |  |  |  |  | - | - |
|  |  | Controls (40% VO2peak) | 0.309 | 0.097 | 12 | 0.095 | Figure 3C | g·min-1 | McArdle vs. Control | Student's T test, unpaired | - | - |
|  |  | McArdle (40% VO2peak) | 0.230 | 0.108 | 9 |  |  |  |  |  | - | - |
|  |  | Controls (50% VO2peak) | 0.288 | 0.138 | 12 | 0.669 | Figure 3C | g·min-1 | McArdle vs. Control | Student's T test, unpaired | - | - |
|  |  | McArdle (50% VO2peak) | 0.313 | 0.125 | 9 |  |  |  |  |  | - | - |
|  |  | Controls (60% VO2peak) | 0.272 | 0.145 | 10 | 0.106 | Figure 3C | g·min-1 | McArdle vs. Control | Student's T test, unpaired | - | - |
|  |  | McArdle (60% VO2peak) | 0.383 | 0.136 | 9 |  |  |  |  |  | - | - |
| **7. Is the fat oxidation rate of McArdle patients higher for the same absolute exercise intensity (workload)?** | Partly, patients showed significantly higher fat oxidation rates (with large effect sizes) at higher workloads. | Controls (0 W) | 0.197 | 0.088 | 12 | 0.149 | Figure 3D | g·min-1 | McArdle vs. Control | Student's T test, unpaired | - | - |
|  |  | McArdle (0 W) | 0.143 | 0.070 | 9 |  |  |  |  |  | - | - |
|  |  | Controls (20 W) | 0.255 | 0.059 | 12 | 0.871 | Figure 3D | g·min-1 | McArdle vs. Control | Student's T test, unpaired | - | - |
|  |  | McArdle (20 W) | 0.249 | 0.094 | 9 |  |  |  |  |  | - | - |
|  |  | Controls (40 W) | 0.273 | 0.099 | 12 | 0.141 | Figure 3D | g·min-1 | McArdle vs. Control | Student's T test, unpaired | - | - |
|  |  | McArdle (40 W) | 0.346 | 0.119 | 9 |  |  |  |  |  | - | - |
|  |  | Controls (60 W) | 0.301 | 0.109 | 11 | **0.026** | Figure 3D | g·min-1 | McArdle vs. Control | Student's T test, unpaired | - | - |
|  |  | McArdle (60 W) | 0.433 | 0.135 | 9 |  |  |  |  |  | - | - |
|  |  | Controls (80 W) | 0.316 | 0.122 | 10 | **0.005** | Figure 3D | g·min-1 | McArdle vs. Control | Student's T test, unpaired | - | - |
|  |  | McArdle (80 W) | 0.511 | 0.140 | 9 |  |  |  |  |  | - | - |
|  |  | Controls (100 W) | 0.288 | 0.153 | 10 | **<0.0001** | Figure 3D | g·min-1 | McArdle vs. Control | Student's T test, unpaired | - | - |
|  |  | McArdle (100 W) | 0.580 | 0.139 | 9 |  |  |  |  |  | - | - |
| **8. Do McArdle's patients who show a decrease in the rate of fat oxidation determined during the last stage of incremental exercise present excess hyperpnea (i.e., increased PetCO2 and VE/VCO2 compared to the first stage)?** | Yes | McArdle Excluded (PetCO2) | 0.835 | 0.105 | 9 | - | Figure 2B | Units relative to first stage | McArdle excluded vs.(McArdle Included vs. Controls) | One-way Anova (Bonferroni-adjusted paired comparison) | - | - |
|  |  | McArdle Included (PetCO2) | 0.988 | 0.081 | 9 | **0.005** |  |  |  |  | - | - |
|  |  | Control (PetCO2) | 1.085 | 0.094 | 12 | **<0.0001** |  |  |  |  | - | - |
|  |  | McArdle Excluded (VE/VCO2) | 1.178 | 0.153 | 9 |  | Figure 2C | Units relative to first stage | McArdle excluded vs.(McArdle Included vs. Controls) | One-way Anova (Bonferroni-adjusted paired comparison) | - | - |
|  |  | McArdle Included (VE/VCO2) | 0.965 | 0.093 | 9 | **0.002** |  |  |  |  | - | - |
|  |  | Control (VE/VCO2) | 0.935 | 0.095 | 12 | **0.0002** |  |  |  |  | - | - |
| **9. Do molecular markers in skeletal muscle tissue differ between McArdle and controls (WT) mice?** | | | | | | | | | | | | |
| 9a.CD36 | No | Controls | 1.330 | 0.960 | 7 | 0.441 | Fig 5 | relative quantification (vs Ponceau) | McArdle vs. Control | Mann Whitney U test | - | - |
|  |  | McArdle | 1.990 | 1.440 | 7 |  |  |  |  |  | - | - |
| 9b.HADH | Yes | Controls | 2.190 | 1.250 | 7 | **0.015** | Fig 5 | relative quantification (vs Ponceau) | McArdle vs. Control | Mann Whitney U test | - | - |
|  |  | McArdle | 0.750 | 0.370 | 6 |  |  |  |  |  | - | - |
| 9c.HSL _total_ | No | Controls | 0.870 | 0.720 | 7 | 0.158 | Fig 5 | relative quantification (vs Ponceau) | McArdle vs. Control | Mann Whitney U test | - | - |
|  |  | McARdle | 2.500 | 2.160 | 7 |  |  |  |  |  | - | - |
| 9d.pHSL_Ser 565_ | No | Controls | 0.730 | 0.410 | 7 | 0.795 | Fig 5 | relative quantification (vs Ponceau) | McArdle vs. Control | Mann Whitney U test | - | - |
|  |  | McArdle | 0.660 | 0.650 | 7 |  |  |  |  |  | - | - |
| 9e.pHSL565/HSL total | No | Controls | 1.100 | 0.520 | 7 | 0.704 | Fig 5 | relative quantification (vs Ponceau) | McArdle vs. Control | Mann Whitney U test | - | - |
|  |  | McArdle | 1.540 | 1.490 | 7 |  |  |  |  |  | - | - |
| 9f.Plin 5 | No | Controls | 0.880 | 0.250 | 7 | 0.373 | Fig 5 | relative quantification (vs Ponceau) | McArdle vs. Control | Mann Whitney U test | - | - |
|  |  | McArdle | 0.720 | 0.190 | 7 |  |  |  |  |  | - | - |
| **10.Do molecular markers in white adipose tissue tissue differ between McArdle and controls (WT) mice?** | | | | | | | | | | | | |
| 10a.AMPK | No | Controls | 1.180 | 0.250 | 5 | 0.471 | Fig 6 | relative quantification (vs Ponceau) | McArdle vs. Control | Mann Whitney U test | - | - |
|  |  | McArdle | 1.830 | 1.570 | 5 |  |  |  |  |  | - | - |
| 10b.HSL _total_ | No | Controls | 0.810 | 0.570 | 6 | 0.928 | Fig 6 | relative quantification (vs Ponceau) | McArdle vs. Control | Mann Whitney U test | - | - |
|  |  | McArdle | 0.990 | 10.400 | 5 |  |  |  |  |  | - | - |
| 10c.ATGL_Ser406_ | No | Controls | 1.980 | 1.260 | 6 | 0.818 | Fig 6 | relative quantification (vs Ponceau) | McArdle vs. Control | Mann Whitney U test | - | - |
|  |  | McArdle | 2.540 | 1.440 | 6 |  |  |  |  |  | - | - |
| 10d.pAMPK _Thr172_ | yes | Controls | 0.730 | 0.220 | 5 | **0.037** | Fig 6 | relative quantification (vs Ponceau) | McArdle vs. Control | Mann Whitney U test | - | - |
|  |  | McArdle | 1.230 | 0.230 | 5 |  |  |  |  |  | - | - |
| 10e.pHSL_Ser565_ | No | Controls | 1.080 | 0.970 | 6 | 0.562 | Fig 6 | relative quantification (vs Ponceau) | McArdle vs. Control | Mann Whitney U test | - | - |
|  |  | McArdle | 1.440 | 1.300 | 6 |  |  |  |  |  | - | - |
| 10f.pHSL_Ser660_ | No | Controls | 1.000 | 0.520 | 6 | 0.689 | Fig 6 | relative quantification (vs Ponceau) | McArdle vs. Control | Mann Whitney U test | - | - |
|  |  | McArdle | 1.340 | 0.720 | 6 |  |  |  |  |  | - | - |
| 10g.Plin5 | No | Controls | 0.620 | 0.890 | 6 | 0.522 | Fig 6 | relative quantification (vs Ponceau) | McArdle vs. Control | Mann Whitney U test | - | - |
|  |  | McArdle | 0.140 | 0.120 | 6 |  |  |  |  |  | - | - |
